# Structural Diversity of Copper(II) Complexes with 9-Deazahypoxanthine and Their *in Vitro* SOD-Like Activity

**DOI:** 10.3390/ijms160715954

**Published:** 2015-07-14

**Authors:** Jana Gáliková, Zdeněk Trávníček

**Affiliations:** Regional Centre of Advanced Technologies and Materials, Department of Inorganic Chemistry, Faculty of Science, Palacký University, 17. listopadu 12, CZ-771-46 Olomouc, Czech Republic; E-Mail: jana.galikova@upol.cz

**Keywords:** copper(II) complexes, 6-oxo-9-deazapurine, 9-deazahypoxanthine, X-ray crystal structure, *in vitro* SOD-like activity

## Abstract

Two structurally different copper(II) complexes of the compositions [{Cu(9dhx)(H_2_O)_3_}_2_(µ-SO_4_)_2_] (**1**) and [Cu(9dhx)_2_(H_2_O)_2_(NO_3_)_2_]·H_2_O (**2**), involving 9-deazahypoxanthine (9dhx; 6-oxo-9-deazapurine; 9-deazahypoxanthine), have been prepared and characterized by elemental analysis, infrared and electronic spectroscopy, electrospray ionisation (ESI) mass spectrometry, thermogravimetric (TG) and differential thermal (DTA) analyses, and cyclic voltammetry. The X-ray structures of complexes **1** and [Cu(9dhx)_2_(H_2_O)_2_(NO_3_)_2_] (**2a**) revealed the distorted octahedral geometry in the vicinity of the copper(II) atoms, with the NO_5_ and N_2_O_4_ donor set, respectively. In the dimeric compound **1**, the {Cu(9dhx)(H_2_O)_3_}_2_ units are bridged by sulfate groups with the Cu···Cu separation being 5.3446(2) Å. In both structures the 9dhx ligands are coordinated through the N3 atoms of the pyrimidine moieties. The SOD-like activity of complexes **1** and **2** was evaluated *in vitro* showing moderate effect, with the IC_50_ values equal to 18.20, and 53.33 μM, respectively.

## 1. Introduction

Copper plays a wide variety of roles in biological systems, mostly involving electron transfer, oxygen binding, and activation of different substrates [1]. Concretely, copper as a catalytic cofactor of various metalloenzymes, e.g., cytochrome c oxidase, tyrosinase or Cu,Zn-superoxide dismutase, participates in many redox and oxygenation reactions [2,3]. Among numerous metalloenzymes, copper, zinc-superoxide dismutase (Cu,Zn-SOD) represents a vital antioxidant in aerobic organisms, which slows down and prevents the oxidative damage by elimination of the superoxide radical, and thus protects cells from damage of biological structures (e.g., lipids, proteins, DNA) induced by reactive oxygen species (ROS) [4,5]. Moreover, excessive generation of ROS is connected with various diseases, such as inflammatory diseases [6], neurodegenerative disorders [7,8], diabetes [9], infections [10,11], and cancer [12]. Due to the unique protective role of Cu,Zn-SOD as an extracellular antioxidant, a variety of low molecular copper-based compounds with SOD-mimic activity, which could be useful in the treatment of various diseases related to superoxide-induced oxidative stress, have been developed and studied to date [13,14].

The potential of structurally varied copper(II) complexes to act as SOD-like active compounds has been verified also by our laboratory. The best SOD-like activity was found for the complex of the composition [Cu_2_(μ-HL^a^)_2_(μ-Cl)_2_Cl_2_] (IC_50_ = 0.253 μM), where HL^a^ = 6-[(2-methoxybenzyl)amino]purine, which showed to be more active than native bovine Cu,Zn-SOD (IC_50_ = 0.480 μM, [15]). The copper(II) complexes, containing methoxy-substituted 6-benzylaminopurine derivatives, were also found to be effective free radical scavengers *in vivo* in the alloxan-induced model of diabetes [16]. Moreover, very interesting results of SOD-like activity were obtained for dimeric perchlorato copper(II) complexes of the general formula [Cu_2_(μ-HL)_4_(ClO_4_)_2_](ClO_4_)_2_ (IC_50_ = 8.67–41.45 μM) containing variously halogeno-substituted 6-benzylaminopurine derivatives (HL) [17]. The copper(II) complexes, involving kinetin (*N*6-furfuryladenine) derivatives, represent another group of compounds with promising SOD-like activity. The best results of *in vitro* SOD-like activity were found for the compounds having the compositions [Cu_2_(μ-HL^a^)_4_Cl_2_]Cl_2_ and [Cu_2_(μ-HL^b^)_2_(μ-Cl)_2_(HL^b^)_2_Cl_2_]·4H_2_O, where HL^a^ = *N*6-furfuryladenine and HL^b^ = *N*6-(5-methylfurfuryl)adenine, with the IC_50_ values equal to 8.13, and 0.71 μM, respectively [18].

Based on these above mentioned promising results, we tried to prepare novel copper(II) complexes showing SOD-like activity, containing 9-deazahypoxanthine-based *N*-donor ligands (6-oxo-9-deazapurines). Such organic ligands were chosen in connection with the fact that 6-oxo-9-deazapurines themselves show various types of biological activities, e.g., they behave as potential purine nucleoside phosphorylase (PNP) inhibitors, possess immunosuppressive ability, which could be beneficial in the treatment of T-cell proliferative and autoimmune diseases [19]. Moreover, ImmunicillinH also known as Forodesine, belonging to the group of the most efficient PNP inhibitors (*C*9-substituted 6-oxo-9-deazapurine derivatives), has already entered the second phase of clinical trials [20]. Furthermore, the first screening of biological activity of zinc(II) compound with 6-oxo-9-deazapurine, *i.e.*, [{Zn(9dhx)(H_2_O)_3_}_2_(μ-SO_4_)_2_], brought promising results of *in vitro* cytotoxicity against the malignant melanoma (G361) cancer cell line (IC_50_ ≈ 21 μM) [21]. Other coordination compounds, *i.e.*, gold(I) and zinc(II) complexes containing 6-(ω-alkyl)oxy-9-deazapurine derivatives (HL_n_), also showed a pharmacological potential. Concretely, the gold(I) complexes of the composition [Au(L_n_)(PPh_3_)] exhibited selective *in vitro* cytotoxicity against some human cancer cell lines, with the best IC_50_ values in a submicromolar range, e.g., 0.6 μM (MCF7) and 0.9 μM (HOS). Moreover, these complexes also significantly influenced the secretion and expression of pro-inflammatory cytokines TNF-α and IL-1β by a similar manner as a commercially used anti-arthritic drug Auranofin [22]. Further, the zinc (II) complexes of the general formula [Zn(HL_n_)_2_Cl_2_]·Solv showed the ability to up-regulate the level of the active form of matrix metalloproteinase-2 (MMP-2) [23].

Taking into consideration these interesting results of biological activities of the above mentioned coordination compounds, the preparation of novel copper(II) coordination compounds containing 6-oxo-9-deazapurine (9-deazahypoxanthine), which could possess interesting SOD-like activity, was the next logical step. Herein, we report the synthesis, characterization and evaluation of SOD-like activity of two structurally different copper(II) complexes of the compositions [{Cu(9dhx)(H_2_O)_3_}_2_(µ-SO_4_)_2_] (**1**) and [Cu(9dhx)_2_(H_2_O)_2_(NO_3_)_2_]·H_2_O (**2**).

## 2. Results and Discussion

### 2.1. Synthesis and General Properties

**Scheme 1:**
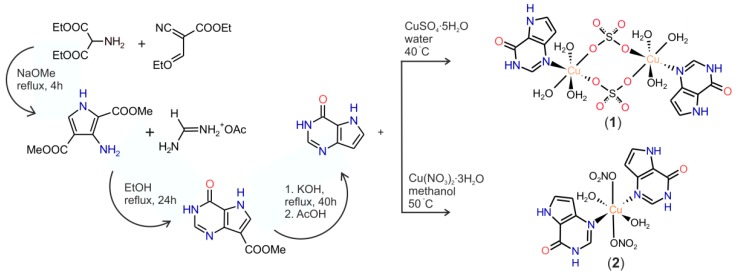
Pathways for the preparation of 6-oxo-9-deazapurine according to a procedure described in [24], and complexes **1** and **2**.

Firstly, 6-oxo-9-deazapurine (9-deazahypoxanthine, 9dhx) was prepared by utilization of the procedure described in the literature [24], which represents a modification of the original synthetic pathway published formerly [25]. The compound was characterized by elemental analysis (C, H, N), FT-IR, ^1^H and ^13^C NMR spectroscopies. Then, the complexes of the compositions [{Cu(9dhx)(H_2_O)_3_}_2_(µ-SO_4_)_2_] (**1**) and [Cu(9dhx)_2_(H_2_O)_2_(NO_3_)_2_]·H_2_O (**2**) were prepared by a single-step synthesis using the reaction of 9dhx with the corresponding copper(II) salt under different reaction conditions, *i.e.*, used reaction media and molar ratios of the starting materials (see Scheme 1). In the case of the preparation of complex **1**, 9dhx was reacted with a significant excess of CuSO_4_·5H_2_O (molar ratio ≈ 1:5) and the reaction was performed in an aqueous solution with pH ~ 5. On the contrary, complex **2** was obtained from the reaction of Cu(NO_3_)_2_·3H_2_O with 9dhx in the ratio of 1:2 in methanol. The composition and purity of the products were proved by elemental analysis (C, H, N), electrospray ionization mass (ESI) spectrometry, FT-IR and UV-Vis spectroscopy, thermogravimetric (TG) and differential thermal (DTA) analyses, and single crystal X-ray analysis.

Complexes **1** and **2** are stable at ambient temperature and could be kept in a desiccator for a long time period without any sign of decomposition. The complexes were found to be soluble in dimethyl sulfoxide (DMSO), *N*,*N*-dimethylformamide (DMF), and alcohols. The molar conductivity values of both complexes were determined for their 10^−3^ M DMSO and DMF solutions, and were found to be 20.0 (**1**) and 64.7 (**2**) S cm^2^·mol^−1^, and 33.1 (**1**) and 40.2 (**2**) S cm^2^·mol^−1^, respectively. It could be anticipated, based on the results of single-crystal X-ray analysis, that both the complexes should behave as non-electrolytes in the solvents used. However, the relatively increased values of the molar conductivity point to the fact that the complexes partially dissociate, and/or that the use of DMSO/DMF as the solvents may lead to the formation of solvolysis species [26]. The probable ligand substitution in DMSO/DMF may logically influence the behavior of the complexes in the solvents used for the study of their physical properties *i.e.*, by UV-Vis spectroscopy (Figure S1 in Supplementary Materials), as well as their SOD-like activity, assessed in DMSO.

### 2.2. TG and DTA Thermal Analyses

With the aim to evaluate the thermal stability of the complexes, both compounds were studied by means of simultaneous TG/DTA analyses, which revealed their decomposition curves (Figure S2 in Supplementary Materials). Complex **1** was found to be thermally stable up to 83 °C. After this temperature, the thermal decomposition of began with the experimental weight loss corresponding to 15.5%, which can be connected with the loss of six coordinated water molecules from the structure of **1** (calc. 15.5% for 6H_2_O). This process, accompanied by an endothermic effect, with a minimum detected at 133 °C on the DTA curve, finished at 159 °C. The further thermal degradation started at 230 °C and proceeded, without formation of thermally stable intermediates, up to *ca.* 700 °C. The most abrupt weight loss was observed between 340 and 374 °C on the TG curve. This decay was associated with an intensive exothermic effect on the DTA curve, with the maximum centred at 367 °C. The final product of the decay appeared at *ca.* 700 °C and it can be associated with the formation of CuO (a total weight loss found/calc. = 77.7/77.2%). In the case of complex **2**, the thermal degradation process began immediately after the start of the analysis at 24 °C, gradually continued without formation of any stable intermediates up to 370 °C, and was associated with a total weight loss of 83.7% (calc. to CuO: 84.5%). In detail, the dehydration of one water molecule of crystallization from the structure of **2** was observed in the first step of thermal degradation, in the range from 24 to *ca.* 150 °C, with the weight loss equal to 3.4% (calc. 3.5% for H_2_O). This dehydration process was accompanied by an endothermic effect, showing the minimum at 37 °C. Subsequently, the thermal decay continued up to *ca.* 370 °C and it was accompanied by the exothermic effects on the DTA curve, with the maxima centered at 254 and 307 °C.

### 2.3. Electrospray Ionization (ESI) Mass Spectrometry

The ESI mass spectra were measured in the positive mode (ESI+) and showed the molecular peak of 9dhx observed at 136.0 *m*/*z*, which clearly confirmed the presence of this organic molecule in the compounds **1** and **2**. The molecular peaks corresponding to complexes **1** and **2** were not detected in the spectra, however, peaks of some fragments were identified. Concretely, the peaks observed in the ESI+ mass spectrum of **1** at 332.1 and 466.8 *m*/*z* may be assigned to [Cu(9dhx)(9dhx–H)]^+^, and [Cu(9dhx)_2_(9dhx–H)]^+^, respectively. Similar fragmentation pattern was observed for the mass spectrum of **2**. The peaks at 260.0, 332.1 and 466.8 *m*/*z* may correspond to the [Cu(9dhx)(NO_3_)]^+^, [Cu(9dhx)(9dhx–H)]^+^, and [Cu(9dhx)_2_(9dhx–H)]^+^ fragments, respectively (Figure S3 in Supplementary Materials).

### 2.4. Infrared Spectroscopy

FT-IR spectra of complexes **1** and **2** measured within the 200–4000 cm^−1^ region were compared with the spectra of free 9dhx. The detected bands at *ca.* 3200, 1685, 1600 and 1553–1468 cm^−1^, corresponding to the ν(N–H)_ar_, ν(C=O), ν(C^…^N)_ring_, and ν(C^…^C)_ring_ vibrations, respectively, unambiguously confirmed the presence of the 9dhx molecule in complexes **1** and **2**. Further, the peaks attributed to the ν(O–H) stretching vibrations were observed at 3484–3262 cm^−1^ [27], indicating the presence of water molecules in both compounds. The strong bands, corresponding to the S–O stretching vibrations of the sulfate group in **1** were found at 1110, 1084, 1021 (ν_3_) and 967 cm^−1^ (ν_1_). For complex **2**, the bands observed at 1445, 1285 and 1023 cm^−1^ could be assigned to the vibrations of the monodentate coordinated NO_3_^−^ group. In the far-IR spectra of **1** and **2**, the peaks detected at *ca.* 350 and 278 cm^−1^ were attributed to the ν(Cu–O)_water,_ and ν(Cu–N) vibrations, respectively. In the spectrum of **1**, the ν(Cu–O)_sulfato_ vibration was observed at 554 cm^−1^ [28].

### 2.5. UV-Vis Spectroscopy

The UV–Vis diffuse-reflectance and solution spectra in DMSO (10^−3^ M) and DMF (10^−3^ M) of complexes **1** and **2** were measured in the 200–1000 nm region. The spectra of **1** and **2** measured in the solid state exhibited broad maxima centred approximately at 820 and 687 nm, respectively, which corresponded to *d*–*d* transitions [29]. The difference in the maxima positions is associated with different coordination environments around the metal centers in **1** and **2**, *i.e.*, with the different donor set and ligand field strength. In the DMSO solution spectra of complexes **1** and **2**, the *d–d* transitions were also observed at 829 (**1**) nm and 811 (**2**) nm, respectively, with the calculated values of molar absorption coefficients equalling 211 (**1**) and 329 (**2**) M^−1^/cm^−1^, while the same transitions were observed at 777 nm (**1**) and 803 nm (**2**), with the molar absorption coefficients equal to 195 (**1**) and 47 (**2**) M^−1^/cm^−1^ in the spectra of DMF solutions. The comparison of the solid-state and solution spectra of **1** showed slight changes in the positions of the maxima (approx. 10 nm in DMSO and 40 nm in DMF). A different situation was found in the spectra of **2**, where the observed maxima were shifted by 124 nm (DMSO) and 116 nm (DMF). Mutual difference between maxima in the solutions may be connected with a bathochromic shift. Aditionally, the differences between the maxima observed in the solid-state and solutions may be connected with the solvent effect on the coordination sphere of the central atom, because DMSO and/or DMF represent the solvents with relatively high values of the donor number and thus the substitution of aqua ligands by DMSO/DMF molecules may occur. However, it seems that the ligand substitution proceeds in the DMSO/DMF solutions of **2** only. The comparison of UV-Vis diffuse-reflectance and DMSO/DMF solution spectra of **1** and **2** is shown in Figure S1 in Supplementary Materials.

### 2.6. Single Crystal X-ray Analysis

The crystals of [{Cu(9dhx)(H_2_O)_3_}_2_(µ-SO_4_)_2_] (**1**) and [Cu(9dhx)_2_(H_2_O)_2_(NO_3_)_2_] (**2a**) suitable for single crystal X-ray analysis were obtained by crystallization of mother liquor of **1** in a closed vial at 40 °C and slow evaporation of a saturated methanol solution of **2**, respectively. Crystal data and structure refinements of complexes **1** and **2a** are summarized in Table 1. Further, bond lengths and angles are given in Table 2, and selected non-covalent contacts are summarized in Tables S1 and S2 (Supplementary Materials).

**Table 1:** Crystal data and structure refinements for **1** and **2a**.

Compound	1	2a
Empirical formula	C_12_H_22_Cu_2_N_6_O_16_S_2_	C_12_H_14_Cu_1_N_8_O_10_
Formula weight	697.56	493.85
Temperature (K)	100(2)	100(2)
Wavelength (Å)	0.71073	0.71073
Crystal system	*Triclinic*	*Triclinic*
Space group	*P*-1	*P*-1
*a* (Å)	6.8164 (2)	8.6756 (5)
*b* (Å)	8.9268 (3)	8.9906 (5)
*c* (Å)	9.2297 (3)	13.0055 (7)
*α* (°)	80.073 (3)	89.127 (5)
*β* (°)	81.919 (3)	74.514 (5)
*γ* (°)	85.943 (3)	61.386 (5)
*V* (Å^3^)	547.08 (3)	850.55 (8)
*Z*, *D_c_* (g·cm^−3^)	1, 2.117	2, 1.924
*F* (000)	354	500
θ range for data collection (°)	3.02 ≤ θ ≤ 25.00	2.89 ≤ θ ≤ 25.00
Reflections collected/unique	5129/1920 (R(int) = 0.0211)	7572/2991 (R(int) = 0.0302)
Data/restraints/parameters	1920/4/190	2991/0/242
Goodness-of-fit on *F*^2^	1.078	1.246
Final *R* indices (*I* > 2σ(*I*))	*R*_1_ = 0.0306, w*R*_2_ = 0.0823	*R*_1_ = 0.0842, w*R*_2_ = 0.2433
*R* indices (all data)	*R*_1_ = 0.0393, w*R*_2_ = 0.0862	*R*_1_ = 0.0879, w*R*_2_ = 0.2444
Largest peak and hole (e·Å^−3^)	0.789, −0.582	1.758, −0.787

**Table 2:** Selected bond lengths and angles (Å, °) in complexes **1** and **2a**. Data for complex **2a** are presented in the following order: 2a/2a^A^, where 2a^A^ represents the second crystallographically independent molecule within the unit cell.

**1**			
Cu(1)–O(4)	1.977(2)	O(4)–Cu(1)–O(3)	169.32(9)
Cu(1)–O(6)	1.980(2)	O(4)–Cu(1)–O(2)	91.56(9)
Cu(1)–O(3)	1.981(2)	O(6)–Cu(1)–N(3)	175.19(9)
Cu(1)–O(2)	2.329(2)	N(3)–Cu(1)–O(2)	91.75(9)
Cu(1)–N(3)	2.018(3)	O(6)–Cu(1)–O(8) ^i^	94.02(8)
Cu(1)–O(8) ^i^	2.428(2)	O(2)–Cu(1)–O(8) ^i^	174.83(7)
**2a/2a^A^**	****	****	****
Cu(1)–O(5)/Cu(1)–O(5) ^i^	1.975(6)	N(3)–Cu(1)–N(3) ^i^/N(3A)–Cu(2)–N(3A) ^ii^	179.999(1)
Cu(1)–N(3)/Cu(1)–N(3) ^i^	2.007(7)	O(2)–Cu(1)–O(2) ^i^/O(2A)–Cu(2)–O(2A) ^ii^	180.000(1)
Cu(1)–O(2)/Cu(1)–O(2) ^i^	2.524(6)	O(5) ^i^–Cu(1)–O(5)/O(5A)–Cu(2)–O(5A) ^ii^	180.000(1)
Cu(2)–O(5A)/Cu(2)–O(5A) ^ii^	1.994(6)	O(5)–Cu(1)–N(3)/O(5A)–Cu(2)–N(3A)	90.2(3)/89.0(3)
Cu(2)–N(3A)/Cu(2)–N(3A) ^ii^	2.031(8)	O(5)–Cu(1)–N(3) ^i^/O(5A) ^ii^–Cu(2)–N(3A)	89.8(3)/91.0(3)
Cu(2)–O(2A)/Cu(2)–O(2A) ^ii^	2.464(8)	O(5)–Cu(1)–O(2)/O(5A)–Cu(2)–O(2A)	81.0(2)/81.5(2)

Symmetry codes for **1** (i) −x + 2, −y, −z + 2; for **2a** (i) −x + 1, −y, −z + 1; (ii) −x, −y, −z.

#### 2.6.1. X-ray Structure of [{Cu(9dhx)(H_2_O)_3_}_2_(µ-SO_4_)_2_] (**1**)

**Figure 1:**
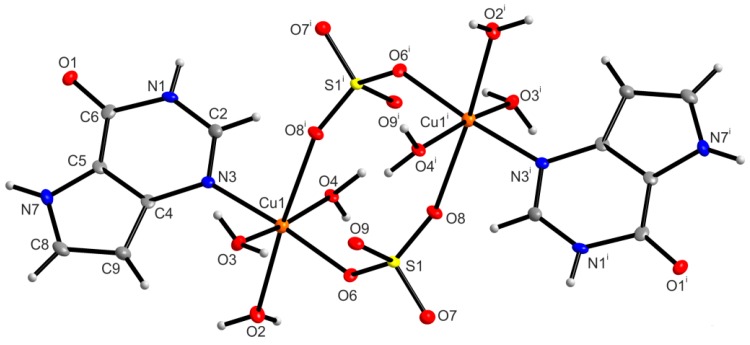
The molecular structure of [{Cu(9dhx)(H_2_O)_3_}_2_(µ-SO_4_)_2_] (**1**), showing the atom numbering scheme. Non-hydrogen atoms are displayed as ellipsoids at the 50% probability level. Symmetry code: (i) −x + 2, −y, −z + 2.

The molecular structure of [{Cu(9dhx)(H_2_O)_3_}_2_(µ-SO_4_)_2_] (**1**) shows that the complex is a centrosymmetric dimer with the Cu···Cu distance being 5.3446(2) Å, where two {Cu(9dhx)(H_2_O)_3_}^2+^ subunits are connected via two sulfato ligands, as depicted in Figure 1. Each copper(II) atom is six-coordinate with the N3 atom from 9dhx, three oxygen atoms from the aqua ligands and two oxygen atoms from the bridging sulfato ligands forming the NO_5_ set [Cu–N3, 2.018(3) Å; Cu–O, 1.977(2)–2.428(2) Å], and adopting the distorted octahedral geometry with the bond angles lying in the range of 83.66(8)–175.19(9)° (Table 2). Based on the search in Crystal Structure Database (CSD ver. 5.36, November 2014) [30], forty-one structures of copper(II) complexes containing bridging sulfate groups were deposited, and only three of these structures represent dimeric compounds and possess the octahedral geometry of the central atom as in **1**. Comparison of the Cu–donor atom bond lengths showed that the Cu–N and Cu–O distances in **1** lie in the range of 1.972(4)–2.023(2) and 1.9527(13)–2.769(2) Å, respectively, found in the molecular structures of the three compounds deposited in CSD.

**Figure 2:**
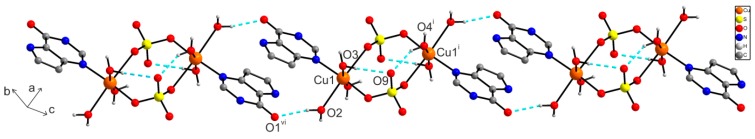
A part of the crystal structure of [{Cu(9dhx)(H_2_O)_3_}_2_(µ-SO_4_)_2_] (**1**), showing the formation of supramolecular polymeric chains, with depicted hydrogen bonds (blue dashed lines) O(3)–H(3W)···O(9), O(4)–H(4W)···O(9) ^i^ and O2–H2W···O1 ^vi^. Hydrogen atoms, except for the hydrogen atoms participating in hydrogen bonds, are omitted for clarity. Symmetry codes: (i) −x + 2, −y, −z + 2; (vi) −x + 1, −y + 1, −z + 1.

**Figure 3:**
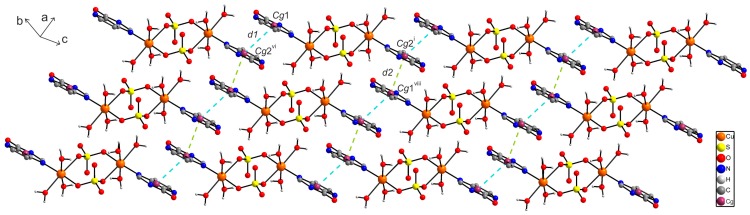
A perspective view of the supramolecular structure of [{Cu(9dhx)(H_2_O)_3_}_2_(µ-SO_4_)_2_] (**1**), showing the different distances of π···π interactions between the centroids of two adjacent and coplanar 9dhx moieties, *d1*{*Cg*1···*Cg*2^vi^} = 3.5277(1) Å (blue dashed lines) and *d2*{*Cg*2^i^···*Cg*1^viii^} = 3.5592(1) Å (green dashed lines). Symmetry codes: (i) −x + 2, −y, −z + 2; (vi) −x + 1, −y + 1, −z + 1; (viii) x, y − 1, z + 1.

In the crystal structure of **1**, individual centrosymmetric dimers are linked via intra- and intermolecular hydrogen bonds of the types O–H···O and N–H···O, and other non-covalent contacts, e.g., π···π and C···C (Table S1 in Supplementary Materials). Particularly, the O2–H2W···O1^vi^ [O···O = 2.808(3) Å; (vi) −x + 1, −y + 1, −z + 1] hydrogen bonds connect the individual dimer units into a polymeric chain as depicted in Figure 2. Moreover, the individual polymeric chains are connected via other hydrogen bonds of the type O–H···O and π···π non-covalent contacts (with the centroid-centroid distances between two adjacent coplanar 9dhx moieties, *Cg*1···*Cg*2^vi^ = 3.5277(1) Å (*d1*); *Cg*2^i^···*Cg*1^viii^ = 3.5592(1) Å (*d2*), (i) −x + 2, −y, −z + 2, (viii) x, y − 1, z + 1) (Figure 3).

#### 2.6.2. X-ray Structure of [Cu(9dhx)_2_(H_2_O)_2_(NO_3_)_2_] (**2a**)

**Figure 4:**
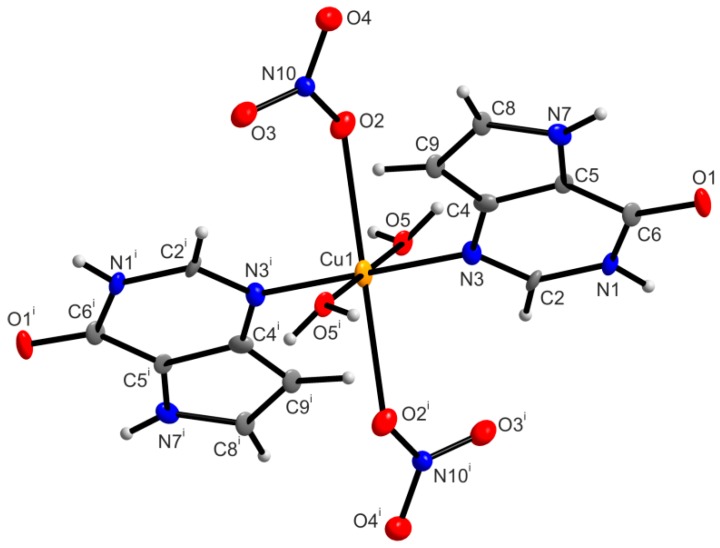
The molecular structure of complex **2a**, together with the atom numbering scheme. Only one of two crystallographically independent molecules of [Cu(9dhx)_2_(H_2_O)_2_(NO_3_)_2_] (**2a**) is shown for clarity. Non-hydrogen atoms are displayed as ellipsoids at the 50% probability level. Symmetry code: (i) −x + 1, −y, −z + 1.

The molecular structure of the mononuclear complex **2a** (Figure 4) consists of two crystallographically-independent molecules of [Cu(9dhx)_2_(H_2_O)_2_(NO_3_)_2_] within the unit cell, further discussed as molecules **2a** and **2a^A^**. Each copper(II) atom is six-coordinate with two N3 atoms of 9dhx, two oxygen atoms from the water molecules and two oxygen atoms from the nitrato ligands [Cu–N, 2.007(7)–2.031(8) Å; Cu–O, 1.975(6)–2.524(6) Å] resulting in the distorted octahedral geometry with the bond angles ranging from 81.0(2)° to 180.0°. With respect to the fact that copper(II) complexes involving the 9-deazapurine molecule have not been reported yet, the bond lengths in the structure of complex **2** were compared with the bond lengths in nine structures deposited in CSD (CSD ver. 5.36, November 2014) [30], containing the purine moiety structurally similar to 9-deazapurine, and having the octahedral geometry around the copper center with the CuN_2_O_4_ chromophore. The given results showed that the Cu–N and Cu–O bond lengths in **2** fell into the range of 1.9035(7)–2.0676(2) and 1.938(2)–2.5399(14) Å, respectively, as was found in the nine molecular structures deposited in CSD.

**Figure 5:**
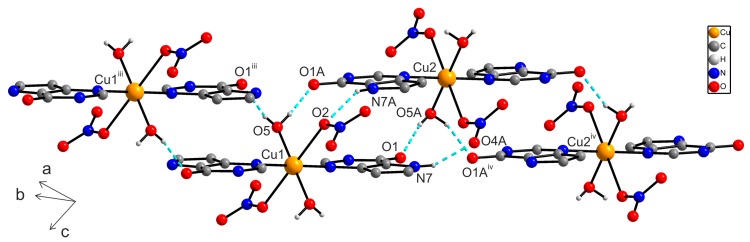
A part of the crystal structure of [Cu(9dhx)_2_(H_2_O)_2_(NO_3_)_2_] (**2a**), showing the formation of a supramolecular polymeric chain formed through intermolecular O–H···O and N–H···O hydrogen bonds (dashed lines). Hydrogen atoms, except for the hydrogen atoms participating in hydrogen bonds, are omitted for clarity. Symmetry codes: (iii) x + 1, y, z; (iv) x − 1, y, z.

**Figure 6:**
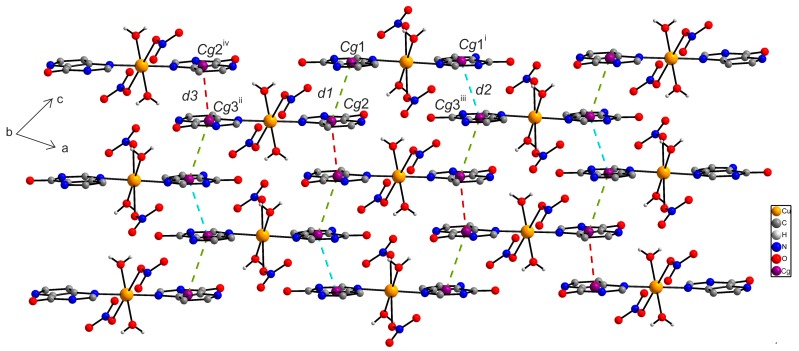
A perspective view (along the *b* axis) of the supramolecular structure of [Cu(9dhx)_2_(H_2_O)_2_(NO_3_)_2_] (**2a**), showing the different distances of π···π interactions between the centroids of two adjacent 9dhx moieties, *d1*{*Cg*1···*Cg*2} = 3.6358(2) Å (green dashed lines), *d2*{*Cg*1^i^···*Cg*3^iii^} = 3.4416(3) Å (blue dashed lines), and *d3*{*Cg*3^ii^···*Cg*2^iv^} = 3.6542(2) Å (red dashes lines). Symmetry codes: (i) −x + 1, −y, −z + 1; (ii) −x, −y, −z; (iii) x + 1, y, z; (iv) x − 1, y, z.

In the crystal structure of **2a**, crystallographically independent molecules **2a** and **2a^A^** are connected via O–H···O and N–H···O hydrogen bonds (Table S2 in Supplementary Materials) forming a one dimensional supramolecular polymeric chain (Figure 5). The individual polymeric chains are further stabilized by a series of non-covalent π···π interactions with the centroid···centroid distances between two adjacent 9-deazapurine moieties equal to *Cg*1···*Cg*2 = 3.6358(2) (*d1*), *Cg*1^i^···*Cg*3^iii^ = 3.4416(3) Å (*d2*) and *Cg*3^ii^···*Cg*2^iv^ = 3.6542(2) Å (*d3*) [(i) −x + 1, −y, −z + 1; (ii) −x, −y, −z; (iii) x + 1, y, z; (iv) x − 1, y, z], and the dihedral angles equal to 0.0(2)° (Figure 6). Additionally, other N–H···O hydrogen bonds also contribute to the stabilization of the crystal structure of **2a**.

### 2.7. In Vitro SOD-Like Activity

*In vitro* SOD-like activity of complexes **1** and **2**, expressed as IC_50_ values, was evaluated by the SOD-like assay carried out by a slightly modified indirect method [15], based on the competitive reaction between complexes **1** and **2**, and 2,3-bis(2-methoxy-4-nitro-5-sulfophenyl)-2*H*-tetrazolium-5-carboxanilide sodium salt [the XTT dye] with potassium superoxide solution in DMSO. The percentage of inhibition of XTT reduction was calculated using the equation, %INH = 100 × (A_b_ − A_s_)/A_b_, where A_b_ (blank) and A_s_ (sample) are absorbances at 470 nm. The IC_50_ values were obtained from the linearized dependence of %INH on a logarithm of molar concentration (first order equation). The SOD-like activity of complexes **1** and **2** was compared to the standard of native bovine Cu,Zn-superoxide dismutase (SOD), with the IC_50_ value equal to 0.480 μM [15].

The results of the SOD-like activity showed a moderate antioxidant effect of complexes **1** and **2**, with the IC_50_ values being 18.20 μM (**1**), and 53.33 μM (**2**), respectively. The calculations were based on the molecular weights of the original complexes. Based on the results, we may say that the activity of complex **1** is rather higher than that of complex **2**. This implies that the SOD-like activity of complexes **1** and **2** depends, among others, on the number of the copper centers.

The comparison of the SOD-like activity was previously described for other monomeric and dimeric copper(II) compounds, where dimeric compounds exhibited lower IC_50_ values of SOD-like activity than the mononuclear ones. For example, dimeric copper(II) complexes of general compositions [Cu_2_(L)(H_2_O)_2_](ClO_4_)_4_ and [Cu_2_(L)Cl_4_] containing tris(2-pyridylmethyl)amine derivatives (L) showed lower IC_50_ values, ranging from 0.54 to 0.76 μM, than the monomeric ones, *i.e.*, [Cu(L)(OH)]ClO_4_, [Cu(L)Cl]ClO_4_ and [Cu(L)](ClO_4_)_2_ (IC_50_ = 5.02–140.0 μM) [31]. The similar situation was found for [Cu(HL^a^)_2_(phen)](ClO_4_)_2_ and [Cu_2_(HL^a^)_2_(L^a^)_2_](ClO_4_)_2_ complexes, where HL^a^ = (*N*-methyl-2-methylol)imidazole, phen = 1,10-phenanthroline, which exhibited the IC_50_ values equal to 0.19 ± 0.01, and 0.10 ± 0.01 μM, respectively [32]. The higher SOD-like activity of the dimeric copper(II) complexes, in comparison with monomeric ones, was also observed by our group. Particularly, the compounds with the general compositions [Cu_2_(μ-HL^b^)_2_(μ-Cl)_4_Cl_2_]Cl_2_ and [Cu_2_(μ-HL^b^)_2_(μ-Cl)_2_Cl_2_] (HL^b^ stands for methoxy substituted 6-benzylaminopurine derivatives) showed the IC_50_ values ranging from 0.253 to 1.250 μM [16]. Further, dinuclear Cu^II^ complexes of the composition [Cu_2_(μ-HL^c^)_4_(ClO_4_)_2_](ClO_4_)_2_ [17] involving halogeno substituted 6-benzylaminopurine derivatives (HL^c^) also showed lower values of IC_50_, ranging from 8.67 to 41.45 μM, than the mononuclear compounds with the general formula [Cu(H_2_O)_2_(L^c^)_2_(phen)]·2MeOH (IC_50_ = 104.1–146.1 μM) containing the same organic ligands (HL^c^) [33]. Generally, it can be assumed that the reason for the higher SOD-like activity of dimeric copper(II) complexes may be assigned to the potential cooperation of both copper(II) atoms in electron transfer and binding of free radicals.

Additionally, the SOD-like activity of the presented dimeric (**1**) and monomeric (**2**) copper(II) complexes was compared with those found for copper(II) compounds with the naturally occurring peptides reported in the literature [34]. In the cited paper, two copper(II) ternary systems with the composition of Cu (II)–HisValHis and Cu (II)–Ac-HisVal-His-NH_2_ exhibited the relative SOD-like activity on a level of 0.6%, and 0.7%, respectively, as compared to the activity of the native Cu, Zn-SOD enzyme (100%). These values are comparable with that obtained for complex **2** with the relative activity of 0.9%. Surprisingly, complex **1**, which does not mimic the structural surrounding of the active site of the native enzyme, revealed significantly higher SOD-like activity (2.6% as compared to the activity of Cu, Zn-SOD) in comparison with the above mentioned Cu (II)-peptide systems.

### 2.8. Cyclic Voltammetry

**Figure 7:**
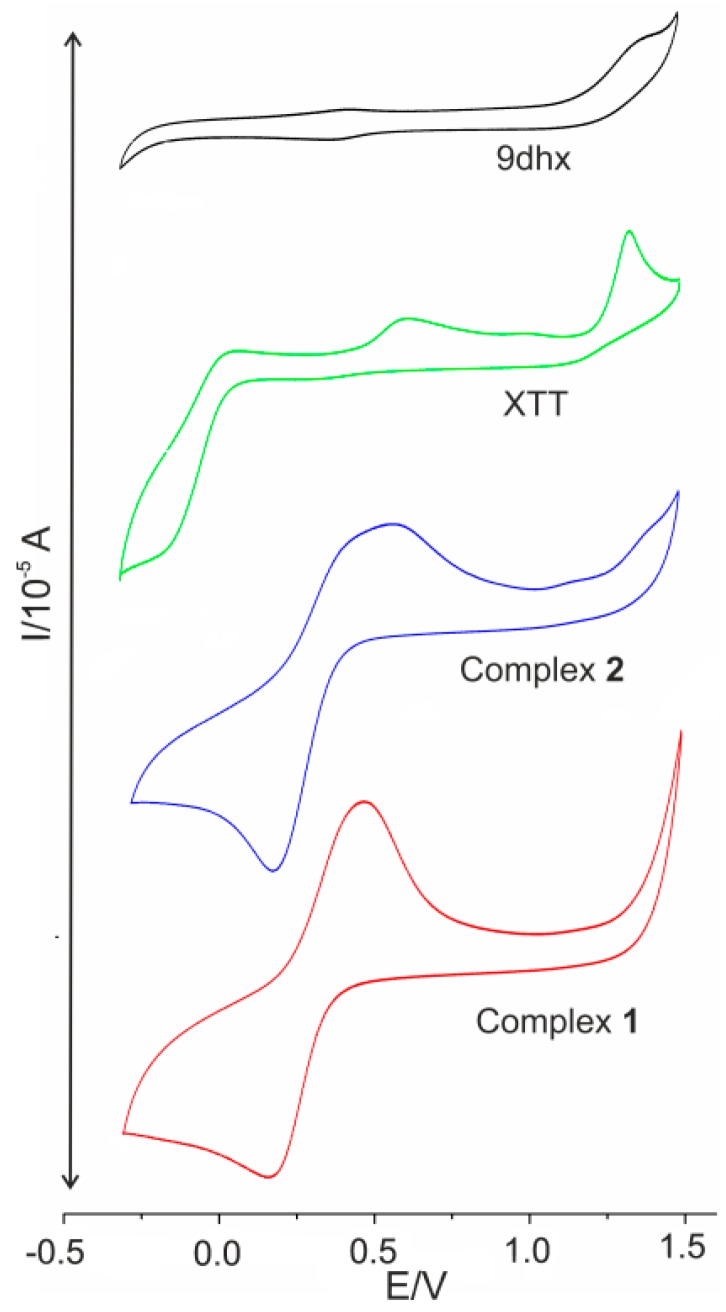
Cyclic voltammograms of 5 mM solutions of complexes **1** (red), **2** (blue), XTT (green) and 9dhx (black) in 0.1 M tetrabutylammonium perchlorate (TBAP) in DMSO (glassy carbon working electrode, potential referred to standard hydrogen electrode (SHE)).

The cyclic voltammetry measurements of complexes **1** and **2** were performed on the solutions keeping similar conditions as those used for the determination of SOD-like activity. The results are summarized in Table 3 and the appropriate cyclic voltammograms are shown in Figure 7. The results of cyclic voltammetry experiments confirmed the quasi-reversible nature of the redox process for both complex **1** and **2**, and therefore supported our assumption that the interaction of the copper(II) complexes with superoxide might proceed as a cyclic redox process. Additionally, the values of the minima of reduction waves obtained for complex **1** and **2**, representing the one-electron reduction of Cu(II) to Cu(I), are much more positive in comparison with the value the corresponding two-electron reduction of XTT and, therefore, the copper(II) complexes are better oxidants as compared to XTT. Hence, the complexes **1** and **2** possess the ability to effectively compete with XTT in the reaction with superoxide.

**Table 3:** Results of cyclic voltammetry measurements.

Compound	DMSO		
	*E*_ox_ *	*E*_red_ *	*I*_p_^a^, *I*_p_^c^
**1**	306	303	−29, 23
**2**	325	326	−22, 29
**XTT**	*E*_ox1_ = 1326 *E*_ox2_ = 614	*E*_red1_ = −137	**

* All the values of potentials (*E*_ox/red_) are given in millivolts (mV) for a scan rate of 200 mV·s^−1^, and all the values of electric current (*I*_p_^a^, *I*_p_^c^) are given in μA.

## 3. Experimental Section

### 3.1. Starting Materials

Chemicals and solvents used for the synthesis of 6-oxo-9-deazapurine (9dhx), complexes **1** and **2** were purchased from the Across Organics Co. (Pardubice, Czech Republic) and Fisher-Scientific Co. (Pardubice, Czech Republic) and were used without any further purification.

### 3.2. Synthesis of [{Cu(9dhx)(H_2_O)_3_}_2_(µ-SO_4_)_2_] (**1**)

A solution of 9dhx (0.5 mmol) in 5 mL water was added to an aqueous solution (3 mL) of CuSO_4_·5H_2_O (2.56 mmol). The final solution (pH ~ 5) was left for crystallization by standing in a closed vessel at 40 °C. After 24 h, nicely shaped blue crystals were filtered off, washed with diethyl ether and dried at 40 °C. Yield: 78% based on Cu. Anal. Calc. for C_12_H_22_N_6_O_16_S_2_Cu_2_ (M_r_ = 697.6): C, 20.7%; H, 3.2%; N, 12.1%; S, 9.2%. Found: C, 20.5%; H, 3.1%; N, 11.7%; S, 9.1%. FT-IR (ν/cm^−1^): 3444 m, 3262 m ν(O–H); 3209 vs ν(N–H)_ar_; 1684 vs ν(C=O); 1590 s ν(C^…^N)_ring_; 1552 m, 1511 m, 1468 m ν(C^…^C)_ring_; 1110 s, 1084 s, 1021 s ν_3_(SO_4_^2−^); 967 m ν_1_(SO_4_^2−^); 554 m (Cu–O)_sulfato_; 350 m (Cu–O)_water_; 278 m ν(Cu–N). ESI+ mass spectra (methanol): *m*/*z* 136.0 [9dhx+H]^+^ (calc. 136.1), 332.1 [Cu(9dhx)(9dhx–H)]^+^ (calc. 332.0), 466.8 [Cu(9dhx)_2_(9dhx–H)]^+^ (calc. 467.1). UV-Vis spectra: λ_max_ (solid state, nm): 820, λ_max_ (10^−3^ M DMSO solution, nm)/ε (M^−1^·cm^−1^): 829/211, λ_max_ (10^−3^ M DMF solution, nm)/ε (M^−1^·cm^−1^): 777/195.

### 3.3. Synthesis of [Cu(9dhx)_2_(H_2_O)_2_(NO_3_)_2_]·H_2_O (**2**)

A solution of 9dhx (0.50 mmol) in 30mL methanol was added to a methanol solution (20 mL) of Cu(NO_3_)_2_·3H_2_O (0.25 mmol). The resulting solution was heated up to 50 °C and stirred for 24 h. Subsequently, the volume of the solution was reduced, and a green product was precipitated from the concentrated solution by diethyl ether. The green solid was filtered off, washed with diethyl ether and dried at 40 °C. The crystals of the composition [Cu(9dhx)_2_(H_2_O)_2_(NO_3_)_2_] (**2a**) were isolated by recrystallization of the powder product of **2** from methanol with the aim to get suitable crystals for single crystal X-ray analysis. Yield: 86% based on Cu. Anal. Calc. for C_12_H_16_N_8_O_11_Cu (M_r_ = 511.8): C, 28.2%; H, 3.2%; N, 21.9%. Found: C, 28.0%; H, 3.1%; N, 22.2%. FT-IR (ν/cm^−1^): 3484 w, 3288 sh ν(O–H); 3205 s ν(N–H)_ar_; 1688 vs ν(C=O); 1603 m ν(C^…^N)_ring_; 1553 m, 1522 m ν(C^…^C)_ring_; 1445 s, 1285 vs, 1023 m ν(NO_3_^−^); 351 m (Cu–O)_water_; 280 m ν(Cu–N). ESI+ mass spectra (methanol): *m*/*z* 136.0 [9dhx+H]^+^ (calc. 136.1), 260.0 [Cu(9dhx)(NO_3_)]^+^ (calc. 260.0), 332.1 [Cu(9dhx)(9dhx–H)]^+^ (calc. 332.0), 466.8 [Cu(9dhx)_2_(9dhx–H)]^+^ (calc. 467.1). UV-Vis spectra: λ_max_ (solid state, nm): 687, λ_max_ (10^−3^ M DMSO solution, nm)/ε (M^−1^·cm^−1^): 811/329; λ_max_ (10^−3^ M DMF solution, nm)/ε (M^−1^·cm^−1^): 803/47.

### 3.4. Methods of Characterization

Elemental analyses (C, H, N) were carried out using a Flash 2000 CHNO-S Analyzer (Thermo Scientific, Waltham, MA, USA). Infrared spectra (FT-IR) were measured on a Nexus 670 spectrometer (Thermo Nicolet, Waltham, MA, USA) in the 150–4000 cm^−1^ (ATR technique) regions. Mass spectra (MS) in the methanol solution (*ca.* 10^−5^ M) were obtained by an LCQ Fleet ion trap mass spectrometer using the electro-spray ionization (ESI) technique (Thermo Scientific, USA). All the observed isotopic distribution representations were compared with the theoretical ones (QualBrowser software, version 2.0.7, Thermo Fischer Scientific, Waltham, MA, USA). Simultaneous thermogravimetric (TG) and differential thermal analyses (DTA) were performed on an Exstar TG/DTA 6200 thermal analyzer (Seiko Instruments Inc., Torrance, CA, USA); ceramic crucible, 150 mL·min^−1^ dynamic air atmosphere, 25–1000 °C temperature range and temperature gradient of 2.5 °C·min^−1^. UV-VIS diffuse-reflectance in nujol suspension and electronic absorption spectra in DMSO (10^−3^ M) and DMF (10^−3^ M) solutions were obtained by a Lambda 35 spectrometer (Perkin-Elmer, Waltham, MA, USA) in the range of 200–1000 nm. The cyclic voltammetry measurements were performed on an electrochemical analyzer CHI600C (CH Instrument Inc., Austin, TX, USA) using a conventional electrochemical, three-electrode-type cell containing an Ag/Ag^+^ reference electrode, a platinum wire auxiliary electrode, and a glassy carbon working electrode. The final potential values referred to SHE were obtained using an internal ferrocene/ferrocenium standard [*E*_1/2_ = 0.680 V *vs*. SHE in DMSO [35]. Conductivity experiments were performed on a Cond 340i/SET (WTW) in DMSO (10^−3^ M) and DMF (10^−3^ M) solutions at 25 °C. The single X-ray data for selected single crystals of [{Cu(9dhx)(H_2_O)_3_}_2_(µ-SO_4_)_2_] (**1**) and [Cu(9dhx)_2_(H_2_O)_2_(NO_3_)_2_] (**2a**) were collected on a four-circle jaxis Xcalibur2 diffractometer (Oxford Diffraction Ltd., UK) equipped with a Sapphire2 CCD detector using MoKα radiation (monochromator Enhance, Oxford Diffraction Ltd., Oxford, UK) and ω-scan technique at 100 K. Data collection and reduction were performed by the CrysAlis software package [36]. The structures were solved by direct methods using SHELX [37] and refined on *F*^2^ using a full-matrix least-squares procedure. H-atoms were located from Fourier maps and refined using a riding model (AFIX 43) in most cases or freely. The N10 and N10a atoms of complex **2** were refined isotropically, only. Molecular graphics were drawn and additional structural parameters were interpreted using *DIAMOND* [38].

### 3.5. SOD-Like Activity Testing

The SOD-like activity of complexes **1** and **2** was evaluated by a slightly modified indirect method according to the procedure published in literature [18].

## 4. Conclusions

In conclusion, this work reports on the synthesis and characterization of two copper(II) compounds of the compositions [{Cu(9dhx)(H_2_O)_3_}_2_(µ-SO_4_)_2_] (**1**) and [Cu(9dhx)_2_(H_2_O)_2_(NO_3_)_2_]·H_2_O (**2**), where 9dhx stands for 6-oxo-9-deazapurine as an *N*-donor ligand. The presented complexes were thoroughly characterized, including the single crystal X-ray analysis, which clearly revealed the structures of the complexes. It has been found that the copper(II) atoms are six-coordinated with the NO_5_ and N_2_O_4_ donor set for **1**, and **2**, respectively. The *in vitro* SOD-like assays showed a moderate SOD-like activity of the presented complexes in comparison with the native bovine Cu,Zn-SOD enzyme (IC_50_ = 0.48 μM), with the IC_50_ values being 18.20 μM (**1**) and 53.33 μM (**2**).

## Supplementary Materials

CCDC No. 1052353 and 1052354 contain the supplementary crystallographic data for **1**, and **2a**, respectively. These data can be obtained free of charge via http://www.ccdc.cam.ac.uk/conts/retrieving.html, or from the Cambridge Crystallographic Data Centre, 12 Union Road, Cambridge CB2 1EZ, UK; fax: (44) 1223-336-033; or email: deposit@ccdc.cam.ac.uk. Supplementary materials can be found at http://www.mdpi.com/1422-0067/16/07/15954/s1.
