# Supplementary material for: Structural Diversity of Copper(II) Complexes with 9-Deazahypoxanthine and Their in Vitro SOD-Like Activity
Source: Int J Mol Sci. 2015 Jul 14;16(7):15954–70. doi: 10.3390/ijms160715954 (PMC4519932; doi:10.3390/ijms160715954)
Supplement: Supplementary file 1 [file ijms-16-15954-s001.pdf]

Supplementary Information

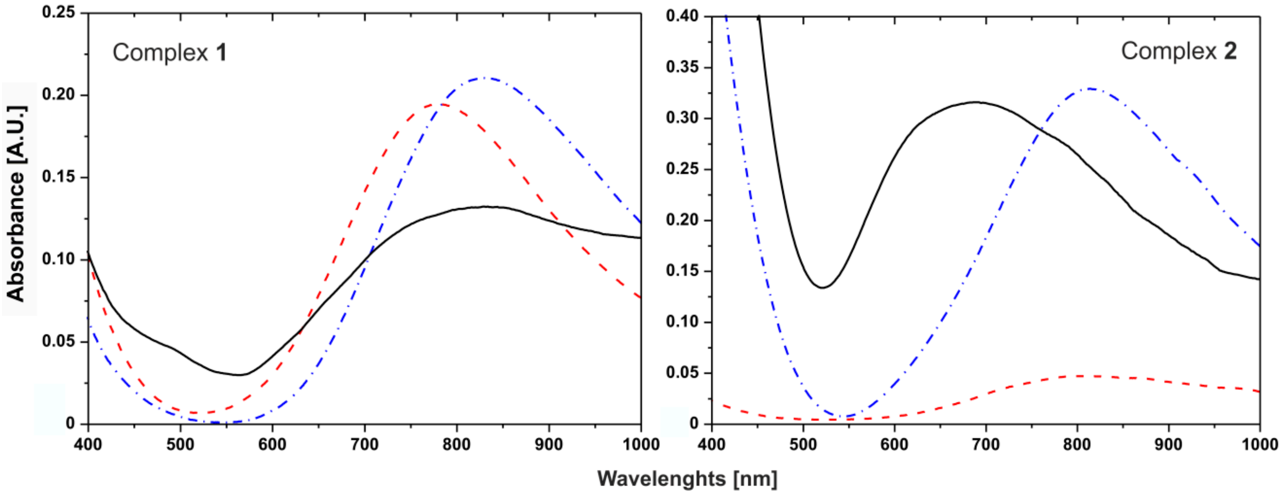

Figure S1. UV-VIS diffuse-reflectance (solid black line), and solution spectra in DMSO (10^{-3} M; dash-dotted blue line) and DMF (10^{-3} M; dashed red line) of complexes **1** (**left**) and **2** (**right**) in the range from 400 to 1000 nm.

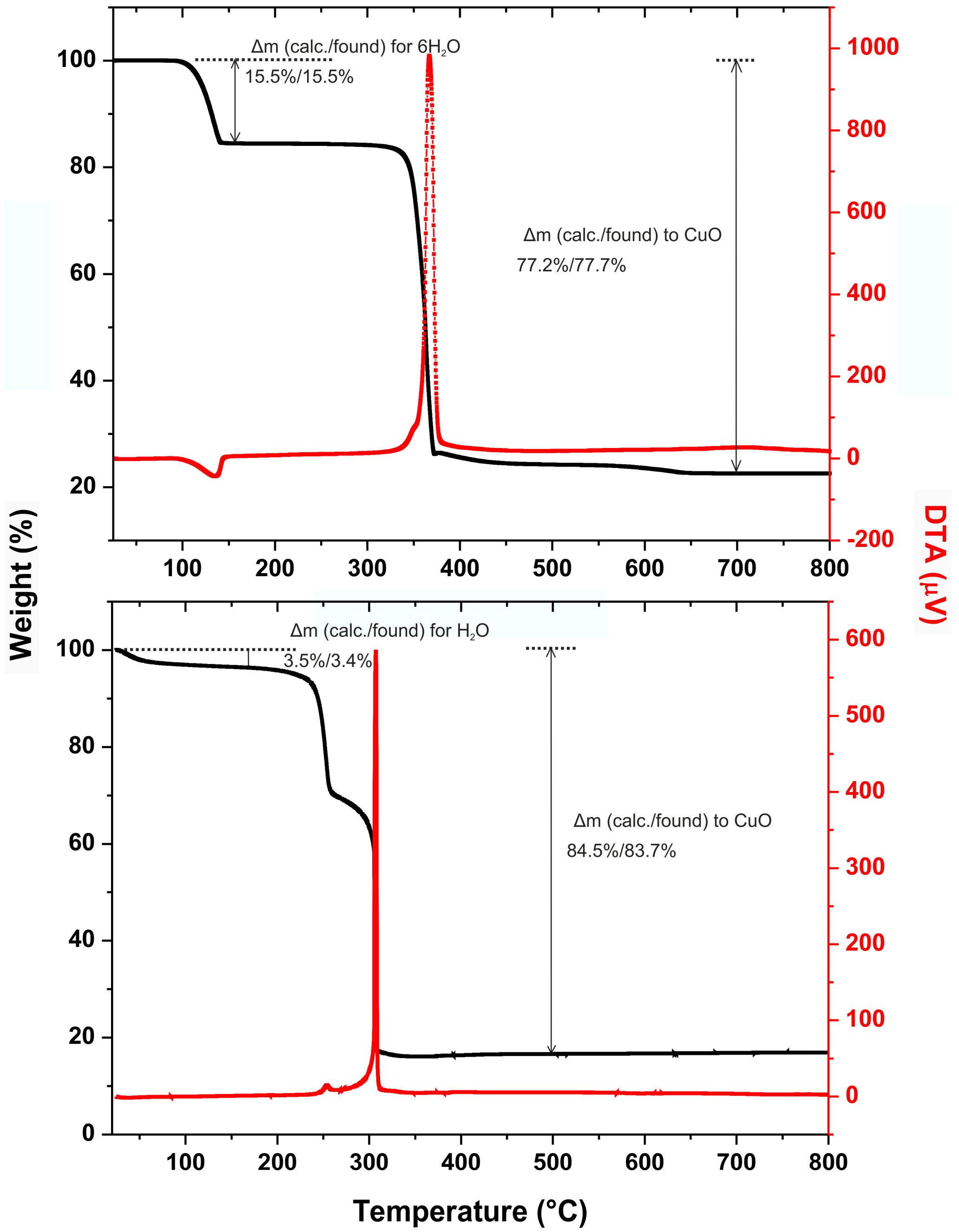

Figure S2. The results of simultaneous TG/DTA thermal analysis of **1** (**up**) and **2** (**down**) showing the TG and DTA curves, and observed and calculated weight losses.

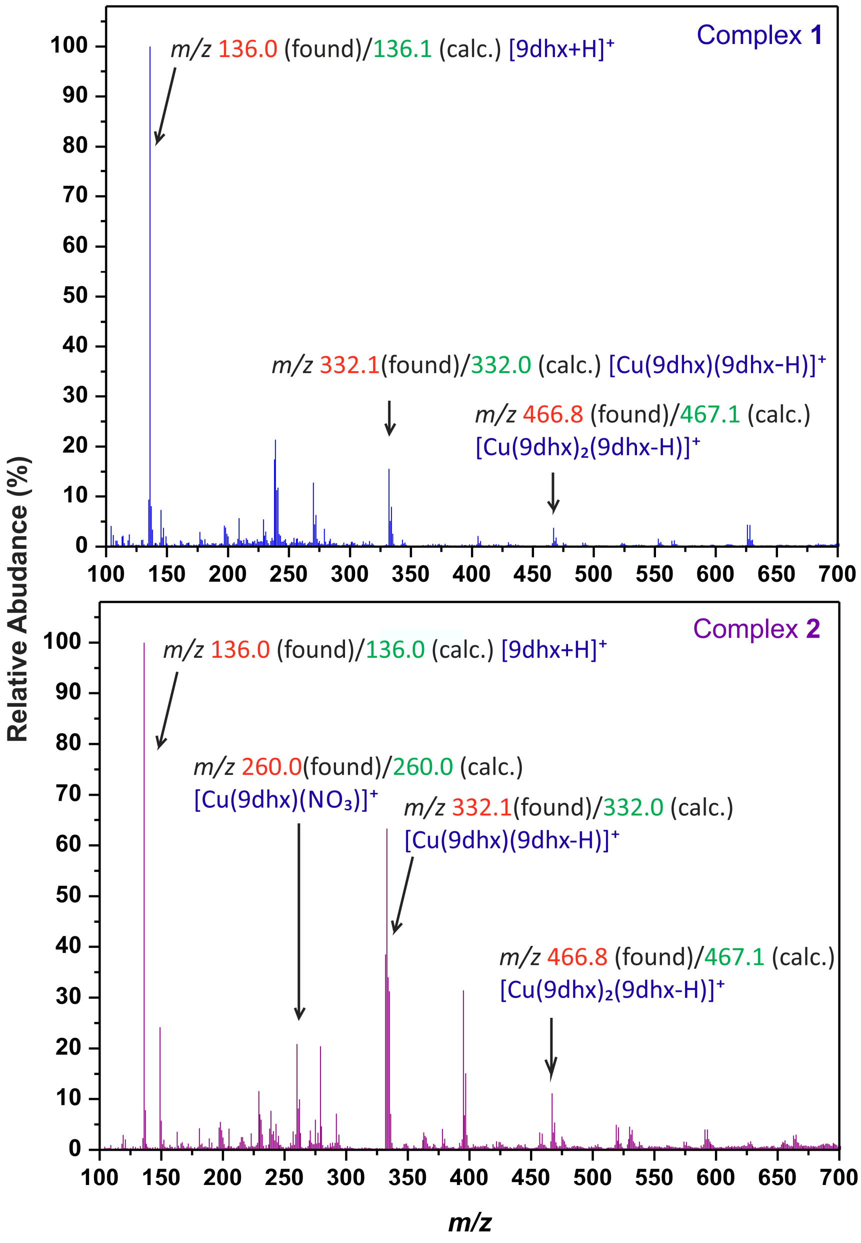

Figure S3. ESI+ mass spectra of **1** (**up**) and **2** (**down**) dissolved in methanol.

Table S1. Selected hydrogen bonds and other non-covalent contacts (Å, °) in the crystal structure of **1** †.

**D−H···A**	***d*** **(D−H)**	***d*** **(H···A)**	***d*** **(D···A)**	**<(DHA)**
O(3)–H(3W)···O(9)	0.86 (3)	2.14 (3)	2.883 (3)	145 (3)
O(4)–H(4W)···O(9) i	0.87 (3)	1.88 (3)	2.685 (3)	153 (3)
N(1)–H(1A)···O(9) ii	0.880 (3)	1.912 (2)	2.774 (3)	166.4 (2)
N(7)–H(7A)···O(7) iii	0.880 (2)	2.131 (2)	2.835 (3)	136.4 (2)
O(2)–H(2V)···O(8) iv	0.87 (3)	2.09 (4)	2.924 (3)	161 (3)
O(3)–H(3W)···O(1) v	0.86 (3)	2.49 (3)	3.041 (3)	123 (2)
O(2)–H(2W)···O(1) vi	0.85 (3)	1.98 (3)	2.808 (3)	163 (3)
O(3)–H(3V)···O(1) vii	0.82 (4)	1.90 (4)	2.717 (3)	174 (4)
O(4)–H(4V)···O(6) iv	0.77 (4)	1.92 (4)	2.683 (3)	170 (4)
C9···C6 vi/C6···C9 vi			3.378 (5)	
C6···C4 vii/C4···C6 vii			3.278 (4)	
*Cg*1···*Cg*2 vi			3.5277 (1)	
*Cg*2 i···*Cg*1 viii			3.5592 (1)	

Symmetry codes: (i) −x + 2, −y, −z + 2; (ii) x, y, z − 1; (iii) x, y + 1, z − 1; (iv) −x + 1, −y, −z + 2; (v) x, y, z + 1; (vi) −x + 1, −y + 1, −z + 1; (vii) −x + 2, −y + 1, −z + 1; (viii) x, y − 1, z + 1; † *Note*: The structural parameters of the non-covalent contacts were interpreted using *DIAMOND*.

Table S2. Selected hydrogen bonds and other non-covalent contacts (Å, °) in the crystal structure of **2a** †.

**D−H···A**	***d*** **(D−H)**	***d*** **(H···A)**	***d*** **(D···A)**	**<(DHA)**
O(5)–H(5W)···O(1A)	0.824 (7)	1.826 (7)	2.646 (10)	172.8 (5)
N(7)–H(7A)···O(4A)	0.880 (10)	1.986 (8)	2.717 (13)	139.6 (6)
O(5A)–H(5X)···O(1)	0.718 (7)	2.065 (7)	2.761 (10)	163.6 (6)
N(7A)–H(7AA)···O(2)	0.881 (10)	2.217 (9)	3.005 (13)	148.7 (6)
O(5)–H(5V)···O(1) iii	0.762 (8)	1.972 (8)	2.721 (11)	167.5 (6)
O(5A)–H(5Y)···O(1A) iv	0.856 (8)	1.919 (7)	2.747 (11)	162.3 (6)
N(1)–H(1A)···O(3) v	0.880 (7)	1.952 (7)	2.765 (10)	152.8 (6)
N(1A)–H(1AA)···O(4A) vi	0.880 (9)	1.964 (8)	2.820 (12)	164.1 (6)
N(1A)–H(1AA)···O(3A) vi	0.880 (9)	2.341 (8)	3.006 (11)	132.5 (5)
C4···C6 vii			3.270 (12)	
C5···C5A			3.431 (13)	
*Cg*1···*Cg*2			3.6358 (2)	
*Cg*1 i···*Cg*3 iii			3.4416 (3)	
*Cg*3 ii···*Cg*2 iv			3.6542 (2)	

Symmetry codes: (i) −x + 1, −y, −z + 1; (ii) −x, −y, −z; (iii) x + 1, y, z; (iv) x − 1, y, z; (v) x − 1, y + 1, z; (vi) x, y + 1, z; (vii) −x, −y, −z + 1; † *Note*: The structural parameters of the non-covalent contacts were interpreted using *DIAMOND*.
